# Lichen Secondary Metabolite Physciosporin Decreases the Stemness Potential of Colorectal Cancer Cells

**DOI:** 10.3390/biom9120797

**Published:** 2019-11-28

**Authors:** Yi Yang, Thanh Thi Nguyen, Iris Pereira, Jae-Seoun Hur, Hangun Kim

**Affiliations:** 1 College of Pharmacy, Sunchon National University, 255 Jungang-ro, Sunchon, Jeonnam 57922, Republic of Korea; yangyi_520@hotmail.com (Y.Y.); thanhbluesky21@gmail.com (T.T.N.); 2 Korean Lichen Research Institute, Sunchon National University, 255 Jungang-ro, Sunchon, Jeonnam 57922, Republic of Korea; jshur1@sunchon.ac.kr; 3 Department of Pharmacology, Chonnam National University Medical School, 160 Baekseo-ro, Dong-gu, Gwangju 61469, Republic of Korea; 4 Faculty of Natural Science and Technology, Tay Nguyen University, Buon Ma Thout 630000, Vietnam; 5 Institute of Biological Sciences, Universidad de Talca, Talca 747-721, Chile; ipereira@utalca.cl

**Keywords:** lichen, *Pseudocyphellaria granulate*, secondary metabolites, physciosporin, cancer stemness inhibition, colorectal cancer cells

## Abstract

Secondary metabolites of lichens are promising bioresources for candidate anti-cancer drugs. Accordingly, several approaches have been proposed for screening these molecules for novel anti-cancer lead compounds. In this study, we found that a non-toxic concentration of physciosporin, a compound isolated from *Pseudocyphellaria granulata*, significantly decreased colony formation on soft agar and spheroid formation by CSC221 cancer stem-like cells. Physciosporin also decreased spheroid formation in other colorectal cancer cell lines, including DLD1, Caco2, and HT29. Aldehyde dehydrogenase-1 (ALDH1), the most important cancer stem marker, was sharply downregulated at both the protein and mRNA level following treatment with physciosporin. Physciosporin also decreased the transcriptional activity of the glioma-associated oncogene homolog zinc finger protein (Gli), as well as the Hes1 and CSL promoters, in reporter assays. Moreover, the drug significantly suppressed spheroid formation in CSC221 cells overexpressing Gli1/2 or ΔEN1 (an S2-cleaved but membrane-tethered form of human Notch1) but did not suppress spheroid formation in cells overexpressing both Gli1/2 and ∆EN1, suggesting that physciosporin suppresses colon cancer cell stemness through the Sonic hedgehog and Notch signaling pathways. Together, these results demonstrate for the first time that physciosporin is a potent inhibitor of colorectal cancer cell stemness.

## 1. Introduction

Cancer can be classified as a genetic disease that alters three types of genes responsible for tumor progression: Oncogenes, tumor suppressor genes, and DNA stability genes. According to this view, cancer develops in a series of steps through the accumulation of molecular changes, progressing from pre-invasive to invasive disease [1,2,3,4,5,6]. Recently, however, the concept of a cancer stem cell was proposed based on our current understanding of oncology and stem cell biology [6,7]. Similar to normal stem cells, cancer stem cells can self-renew, have extensive slow proliferative potential, and give rise to new phenotypically diverse progeny with variable proliferative potential. It has been suggested that cancer stem cells persist in tumors as a distinct population and cause relapse and metastasis by giving rise to new tumors [6,8,9,10]. Therefore, the cancer stem cell model helps explain the failures of many cancer therapies and highlights deficiencies in certain research approaches [1,4,5,7,11,12,13,14,15,16]. Currently, a new model of development of tumors is emerging in which tumors, like normal adult tissues, arise from stem cells. In this model, cancer stem cells (CSCs) are the real driving force behind tumor growth. CSCs share all of the fundamental traits of stem cells: Self-renewal by asymmetric division, reduced proliferation and differentiation, and resistance to apoptosis [17,18,19,20]. Therefore, therapeutic approaches are being developed to block embryonic pathways that play roles in CSCs, including the Notch, Hedgehog, and Wnt/β-catenin pathways [3,17,18,19,20,21,22]. In the Notch signal-receiving cell, once notch ligands Jagged and Delta bind to Notch receptors, γ-secretase releases the intracellular domains of the Notch receptors (NICD) from the transmembrane through proteolytic cleavage (S3 cleavage). NICD associates with the CSL (CBF1/Su(H)/Lag-1) transcription factor complex after nuclear translocation, resulting in subsequent activation of HES-family members, like Hes1 (hairy and enhancer of split-1), and other downstream target genes [20,23]. In the Hedgehog (Hh) signaling pathway, zinc-finger transcription factors of the Gli family, like Gli1 and Gli2, serve as key transcription factors in the mediation of signal to the nucleus [24]. Therefore, suppression of these transcription factors is considered to be an effective strategy for cancer stemness inhibition.

Lichen is a novel resource for new anti-cancer chemicals [25]. In a previous study, we showed that the lichen-derived compound physciosporin inhibits the cell motility of lung cancer cells and suppresses proliferation, motility, and tumorigenesis in colorectal cancer cells [26,27]. To further characterize the pharmacological properties of physciosporin, we evaluated its cytotoxicity, as well as its effects on spheroid formation, colony formation, and expression of cancer stemness-related proteins, genes, and transcription factors in human colorectal CSCs. Finally, we showed that physciosporin suppressed colon cancer cell stemness through the Sonic hedgehog and Notch signaling pathways, suggesting that the drug is a potent inhibitor of colorectal cancer cell stemness.

## 2. Materials and Method

### 2.1. Lichen Collection

*Pseudocyphellaria granulata* (CL130259) growing on the trunk of on *Nothofagus* sp. was collected at Lago Anibal Pinto (52°01′30.8″ S, 72°22′38.8″ W, alt. 190m), Parque Nacional Torres del Paine, Patagonia, Chile on 20 January 2013 during field trips organized by Prof. Iris Pereira at Talca University. The permit to collect lichen specimens from this location was issued by the Administration of the National Forestry Corporation (CONAF) of Punta Arenas and the Administration of the National Park Torres del Paine, Magallanes Region and Chilean Antarctic. Field studies did not involve any endangered or protected species. Duplicates were deposited at the Korean Lichen and Allied Bioresource Center (KOLABIC) in the Korean Lichen Research Institute (KoLRI), Sunchon National University, Korea.

### 2.2. Extraction, Separation, and Identification of Physciosporin

*Pseudocyphellaria granulata* (CL130259) extract was separated by high-performance liquid chromatography (HPLC) as described [28]. Briefly, elution was performed with a solvent system of methanol:water:phosphoric acid (80:20:1, *v*/*v*/*v*) at a column temperature of 40 °C. Physciosporin fraction was collected and then purified by preparative thin-layer chromatography (TLC) method using 5% methanol/dichloromethaneand, and the single peak was confirmed by liquid chromatography-mass spectrometry (LC-MS, Agilent Technologies, Santa Clara, CA, USA) after purification. The structure of physciosporin was confirmed by nuclear magnetic resonance (NMR) analysis [27].

### 2.3. Cell Culture

The human cancer stem cell line CSC221 [29], human colon cancer cell lines DLD1 and HT29, and HEK293T human embryonic kidney cells were maintained in Dulbecco′s modified eagle′s medium (DMEM) (GenDEPOT, Katy, TX, USA) supplemented with 10% fetal bovine serum (FBS) and 1% penicillin–streptomycin solution under 5% CO_2_ in a humidified atmosphere at 37 °C.

### 2.4. MTT Assay

Cells were seeded at a density of 2.5 × 10^3^ cells/well in 96-well plates, grown overnight, and then treated with crude acetone extracts of *Pseudocyphellaria granulate (P. granulate)* or pure physciosporin for 48 h. All samples were dissolved in dimethyl sulfoxide (DMSO) and diluted with DMEM medium to obtain the indicated concentrations. 3-(4,5-dimethylthiazol-2-yl)-2,5-diphenyltetrazolium bromide (MTT) was added and incubated with the cells for 4 h after treatment. After formazan crystals were dissolved in DMSO, absorbance at 540 nm was determined using a microplate reader with the Gen5 (2.03.1) software (BioTek, Winooski, VT, USA).

### 2.5. Reporter Assay

HEK293T cells were transfected with TOPFLASH-luc-, Gli-luc-, Hes-1-, and CSL-conjugated firefly plasmid along with *Renilla*-luc (pRL-TK) plasmid using the X-treme GENE 9 DNA transfection reagent (Roche, Werk Penzberg, Germany). Twelve hours after transfection, cells were treated with crude acetone extract of *P. granulata*, physciosporin, or DMSO (0.01%) after 12 h transfection and were incubated for 48 h at 37 °C under 5% CO_2_. Luciferase activity was measured using the Dual-Luciferase reporter assay kit (Promega, Madison, WI, USA), normalizing firefly against *Renilla* luciferase activity to control for transfection efficiency.

### 2.6. Soft Agar Colony-Formation Assay

A bottom layer of agar (0.5%) in complete DMEM (Gibco, NY, USA) media was poured and allowed to solidify, followed by an upper layer (0.35%) containing 2.5 × 10^3^ CSC221 cells suspended in DMEM medium–agar mixture. Cells were fed twice per week with cell culture media containing crude acetone extract of *P. Granulata*, physciosporin, or DMSO (0.01%). After 2 weeks of incubation, colonies were counted, and relative colony-formation ability was determined based on the pixel intensity of the colony area using the IMT iSolution software (IMT iSolution Inc., Northampton, NJ, USA) from random microscope fields on each plate. Colony-formation ability served as an indicator of the malignancy of tumor cells. Data are presented as the average of three independent experiments.

### 2.7. Spheroid Assay

Trypsinized resuspended cells were rinsed with N2-supplemented DMEM/F12 (Invitrogen, Carlsbad, CA, USA), containing 20 ng/mL human recombinant epidermal growth factor (hrEGF; Biovision, Atlanta, GA, USA) and 10 ng/mL human basic fibroblast growth factor (hbFGF; Invitrogen). Cells (5 × 10^3^/well) were seeded in ultra-low attachment 24-well plates (Corning Inc., Corning, NY, USA), and then treated with crude acetone extract of *P. granulata*, physciosporin, or DMSO (0.01%) 3 h after seeding. The cells were then incubated for 14 days at 37 °C under 5% CO_2_. The images of sphere were taken by inverted phase-contrast microscopy (Nikon, Kawasaki, Japan), and the relative sphere formation ability was calculated through the IMT iSolution software (IMT iSolution Inc., Northampton, NJ, USA) measuring the pixel intensity of the sphere area randomly in each plate. Data are presented as the average of three independent experiments.

### 2.8. Western Blotting

Cells treated with physciosporin for 48 h were washed twice with ice-cold phosphate-buffered saline (PBS) and lysed in lysis buffer [30]. Antibodies against ALDH1 (sc-166362; Santa Cruz Biotechnology, Dallas, TX, USA), CD133 (CA1217; Cell Applications, San Diego, CA, USA), CD44 (3570; Cell Signaling Technology, Danvers, MA, USA), Lgr-5 (ab75850; Abcam, Cambridge, MA, USA), and Msi-1 (ab52865, Abcam) were used to detect stemness factors. α-tubulin (2125, Cell Signaling Technology) antibody was used as an internal control. The luminescence photon from proteins were detected by an Image Quant LAS 4000 mini using horseradish peroxidase-conjugated secondary antibody (Thermo Fisher Scientific, Waltham, MA, USA) with an Immobilon Western Chemiluminescent HRP Substrate Kit (Merck Millipore, Billerica, MA, USA). Quantitation of bands were performed by using Multi-Gauge 3.0 software, and the relative density of each band was calculated based on the density of the internal control bands in each sample. Values are shown as arbitrary densitometric units corresponding to signal intensity. All results are representative of at least three independent experiments.

### 2.9. Quantitative Reverse-Transcription PCR (qRT-PCR)

Quantitative RT-PCR (qRT-PCR) was conducted as previously described [31]. Briefly, total RNA (1 mg) isolated from DMSO-, *P. granulata*-, or physciosporin- treated CSC221 cells using RNAiso Plus (TaKaRa, Otsu, Japan) was converted to cDNA using the M-MLV reverse transcriptase kit (Invitrogen). qPCR was performed using SYBR Green (Enzynomics, Seoul, Korea). Primers used for real-time PCR were as follows: *ALDH1* (forward) 5′-tgttagctcatgccgacttg-3′ and (reverse) 5′-ttcttagcccgctcaacact-3′; *CD133* (forward) 5′-ggacccattggcattctc-3′ and (reverse) 5′-caggacacagcatagaataatc-3′; *CD44* (forward) 5′-tgccgctttgcaggtgtat-3′ and (reverse) 5′-ggcctccgtccgagaga-3′; *Lgr5* (forward) 5′-ctcttcctcaaaccgtctgc-3′ and (reverse) 5′-gatcggaggctaagcaactg-3′; *Msi-1* (forward) 5′-accaagagatccaggggttt-3′ and (reverse) 5′-tcgttcgagtcaccatcttg-3′; *Bmi-1* (forward) 5′-cca gggcttttcaaaaatga-3′ and (reverse) 5′- ccgatccaatctgttctggt-3′; *EphB1* (forward) 5′-tgcaag gagaccttcaacct-3′ and (reverse) 5′- cggtgttgattttcatgacg-3′; *Hes-1* (forward) 5′-ctgaagaaagat agctcgcg-3′ and (reverse) 5′-acttccccagcacactt-3′; *Gli-1* (forward) 5′-ccatacatgtgtgagcacga- 3′ and (reverse) 5′-ggcacagtcagtctgcttt-3′; *Gli-2* (forward) 5′-caacgcctactctcccagac-3′ and (reverse) 5′- gagccttgatgtactgtaccac-3′; *SMO* (forward) 5′-catccctgactgtgagatca-3′ and (reverse) 5′-caccatcttggtgacatgct-3′; and *GAPDH* (forward) 5′-atcaccatcttccaggagcga-3′ and (reverse) 5′-agttgtcatggatgaccttggc-3′. qRT-PCR reactions and analyses were performed on a CFX instrument (Bio-Rad, Hercules, CA, USA).

## 3. Results

### 3.1. Physciosporin, a Major Secondary Metabolite of Pseudocyphellaria Granulata, Exerts Anti-Cancer Activity Against the Human Colon Cancer Stem Cell Line CSC221

**Figure 1:**
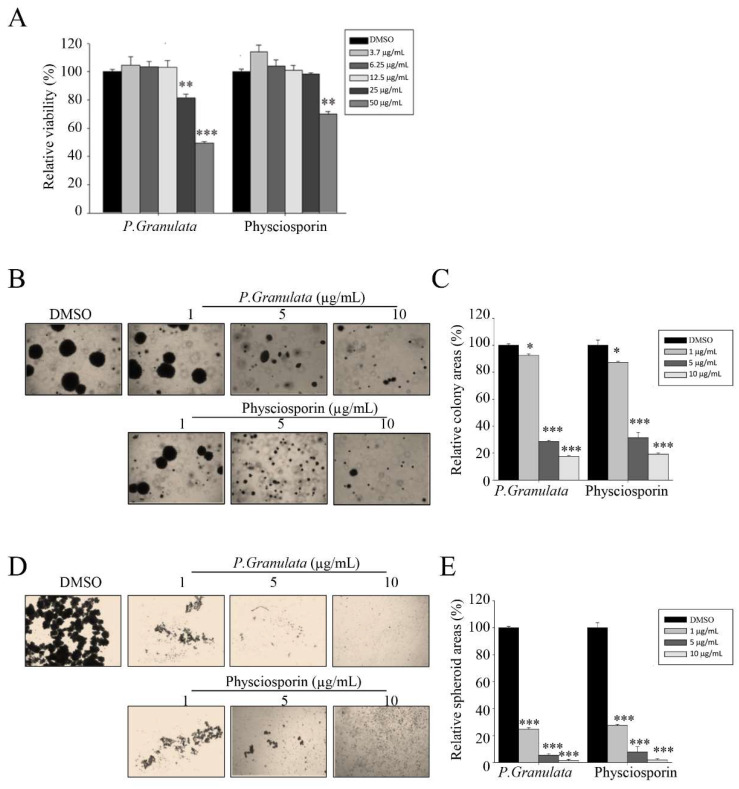
Acetone extracts of *Pseudocyphellaria granulata* and physciosporin inhibit CRC221 cell stemness. (**A**) Relative viability of cells treated with acetone extract of *P. granulata* or pure physciosporin. CSC221 cells were treated with crude extract or physciosporin at concentrations ranging from 3.7 to 50 µg/mL for 48 h, and cell viability was measured by MTT assay. (**B**,**C**) Soft agar colony-formation assay of CSC221 cells treated with *P. granulata* crude extract or physciosporin (**B**), and quantification of the percent colony area in each group (**C**). (**D**,**E**) Representative images of spheroid formation of CSC221 cells treated with *P. granulata* crude extract and single-compound physciosporin for 14 days (**D**), and quantitative analysis of the number of spheroids following each treatment (**E**). Quantitative data were obtained from three independent experiments (*n* = 3). Data are means ± standard error of the mean (SEM), and statistical analysis was performed by one-way ANOVA. * *p* < 0.05; ** *p* < 0.01; *** *p* < 0.001 vs. CSC221 cells treated with DMSO.

*P. granulata* has been used as a representative species in lichen taxonomy study containing the main secondary metabolites of physciosporin [32,33]. In a previous work, we showed that physciosporin inhibits the cell motility of lung cancer cells and suppresses proliferation, motility, and tumorigenesis of colorectal cancer cells [26,27]. To further characterize the pharmacological properties of physciosporin, we isolated the compound from *P. granulata* and purified it using the method described in our previous study [27]. We then conducted MTT assays on CSC221 human colorectal adenocarcinoma-enriched cancer stem cells treated with crude acetone extract of *P. granulata* or physciosporin. The crude extract exerted much more cytotoxicity than physciosporin at 25 and 50 µg/mL (Figure 1A). To evaluate the effects of the drug on in vitro proliferation, differentiation, and self-renewal capacity of stem clones of CSC221, we performed soft agar colony-formation assays on CSC221 cells treated with acetone crude extract or physciosporin at sublethal doses (non-toxic concentrations: 1, 5, or 10 µg/mL). The quantitative data revealed that the crude acetone extract of *P. granulata* and physciosporin significantly decreased colony formation in a dose-dependent manner (Figure 1B,C).

In addition, to monitor the effects of the drug on the differentiation and self-renewal capacity of CSC221 cells, we performed spheroid formation assays. As shown in Figure 1D,E, crude acetone extract of *P. granulata* and physciosporin both sharply decreased spheroid formation in a dose-dependent manner. Taken together, these findings show that acetone extract of *P. granulata* extract, as well as the pure secondary metabolite physciosporin, significantly inhibited the proliferation, differentiation, and self-renewal ability of CSC221 colon cancer stem cells at sublethal doses.

### 3.2. Acetone Crude Extract of Pseudocyphellaria granulata and Physciosporin Inhibit Spheroid Formation of Colorectal Cancer Cells

**Figure 2:**
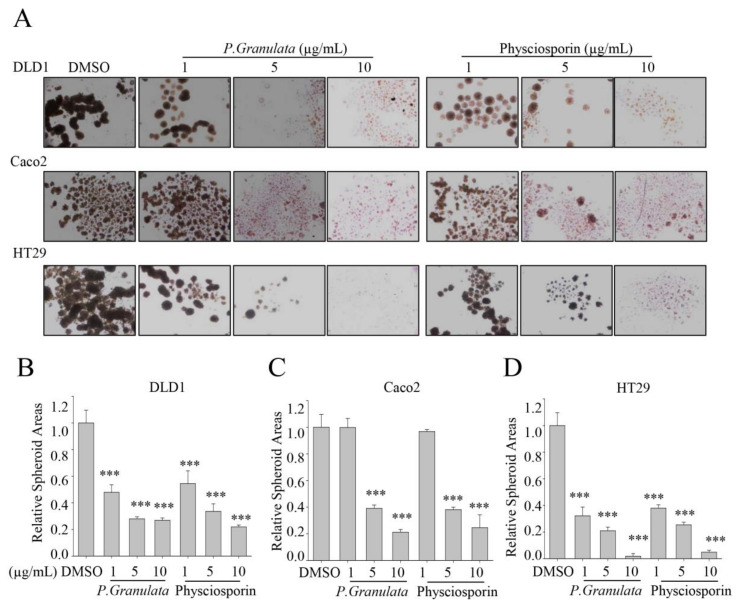
Acetone extracts of *Pseudocyphellaria granulata* and physciosporin inhibit colorectal cancer (CRC) cell stemness. (**A**) Representative images of spheroid formation of DLD1, Caco2, and HT29 cells treated with *P. granulata* crude extract or physciosporin for 14 days. (**B**–**D**) Quantitative analysis of relative spheroid number in DLD1 (**B**), Caco2 (**C**), and HT29 (**D**) cultures. Quantitative data were obtained from three independent experiments, *n* = 3. Data are means ± standard error of the mean (SEM), and statistical analysis was performed by one-way ANOVA. *** *p* < 0.001 vs. DMSO-treated CRC cells.

To further confirm the ability of crude extract of *P. granulata* and physciosporin to inhibit colorectal cancer (CRC) stemness, we expanded the spheroid assay to include three CRC cell lines: DLD1, Caco2, and HT29. As shown in Figure 2A, fewer spheres formed in the DLD1 and HT29 cultures treated with crude extract of *P. granulata* and with physciosporin compared with those treated with DMSO (negative control), at all treatment concentrations. However, crude extract of *P. granulata* and physciosporin did not inhibit spheroid formation by Caco2 cells at 1 µg/mL, although they did exert inhibitory activity at two higher concentrations (Figure 2A). The quantitative data yielded consistent results: Both crude extracts of *P. granulata* and physciosporin suppressed spheroid formation in a dose-dependent manner in all three CRC cells (Figure 2B–D). Taken together, these results suggested that physciosporin indeed inhibits the cancer stemness of CRC cells.

### 3.3. Acetone Crude Extract of Pseudocyphellaria granulata and Physciosporin Reduced Cancer Stemness-Related Protein and Gene Expression

**Figure 3:**
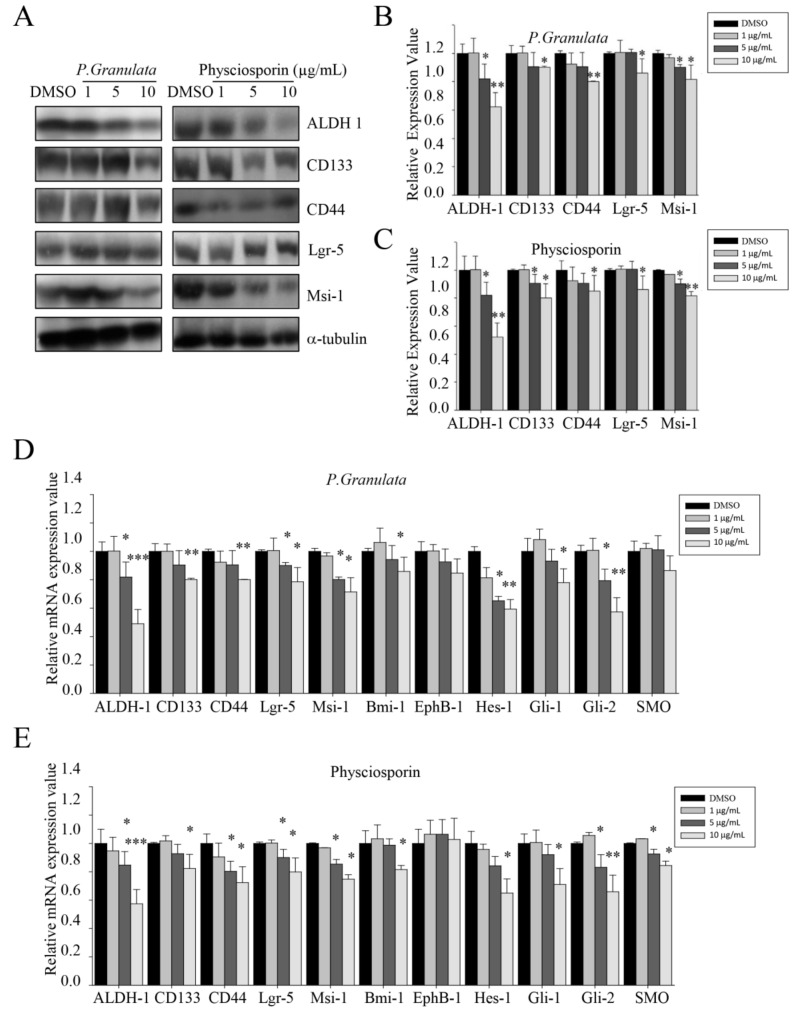
Effects of crude extracts of *Pseudocyphellaria granulata* and physciosporin on cancer stemness-related protein and gene expression. (**A**) Western blot analysis of cancer stem cell markers aldehyde dehydrogenase-1 (ALDH1), cluster of differentiation 133 (CD133), CD44, Lgr5, and Musashi-1 in CSC221 cells treated with acetone extracts of *P. granulata* or various concentrations of physciosporin. (**B**,**C**) Quantitative analysis of the protein expression levels of cancer stem cell markers ALDH1, CD133, CD44, Lgr5, and Msi-1 in CSC221 cells treated with various concentrations of acetone extracts of *P. granulata* (**B**) or physciosporin (**C**). (**D**,**E**) Relative mRNA expression of cancer stemness markers *ALDH1*, *CD133*, *CD44*, *Lgr5*, *Msi-1*, *Bmi-1*, *EphB-1*, *Hes-1*, *Gli-1*, *Gli-2*, and *SMO* on CSC221 cells after treatment with various concentrations of acetone extract of *P. granulata* (**D**) and physciosporin (**E**). Quantitative data were obtained from at least two independent experiments. Data are means ± standard error of the mean (SEM), and statistical analysis was performed by one-way ANOVA. * *p* < 0.05; ** *p* < 0.01; *** *p* < 0.001 vs. DMSO-treated CSC221 cells.

To determine whether physciosporin affects the expression of cancer stemness markers in CRC cells, we monitored the protein expression level of aldehyde dehydrogenase-1 (ALDH1), cluster of differentiation 133 (CD133), CD44, Lgr5, and Musashi-1 (Msi-1) in CSC221 cells. As shown in Figure 3A–C, the ALDH1 level was decreased by around 50% in cells treated with either crude extract of *P. granulata* or physciosporin at 10 µg/mL. Other cancer stem cell markers were slightly reduced at the same concentration; the quantitative data revealed that the change was significant for all such markers. Many more changes in cancer stemness-related gene expression were detected by qRT-PCR assay. Consistent with the protein measurements, *ALDH1* mRNA expression level was downregulated by as much as 50% by both crude extract of *P. granulata* and physciosporin at high concentrations (Figure 3D,E). Similarly, the mRNA levels of *Hes1* and *Gli2* were decreased by around 40% by both crude extract and purified drug at a concentration of 10 µg/mL. Other cancer stemness-related genes, except for *EphB-1* and *SMO*, were downregulated to different extents by 20% to 30%, by extract or physciosporin at 10 µg/mL. By contrast, the mRNA level of *EphB-*1 did not change significantly under these conditions, and the *SMO* mRNA level was reduced only slightly at concentrations of 5 and 10 µg/mL. Together, these findings show that acetone crude extract of *P. granulata* and physciosporin can decrease cancer stemness-related protein and gene expression.

### 3.4. Crude Acetone Extract of Pseudocyphellaria granulata and Physciosporin Affected Cancer Stemness-Related Signaling Pathways

**Figure 4:**
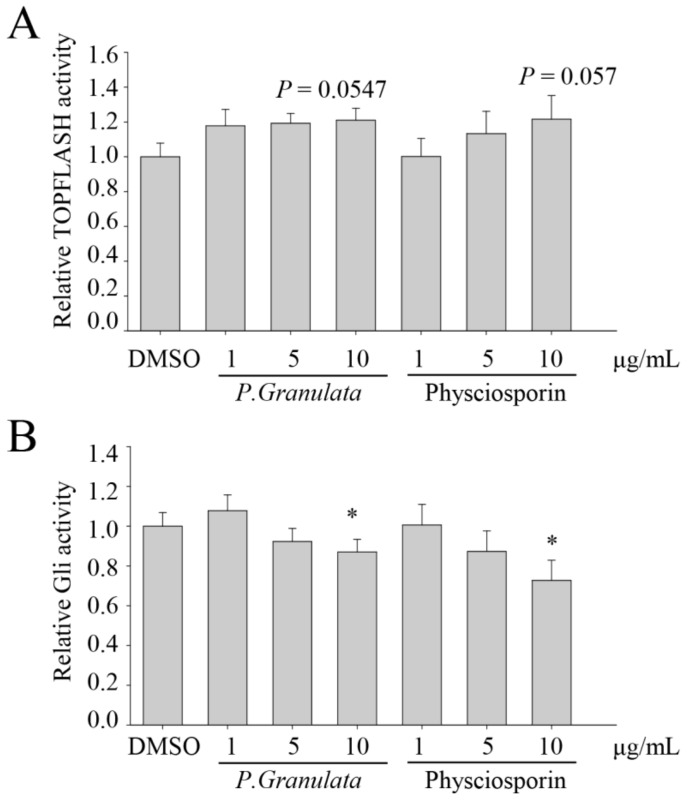

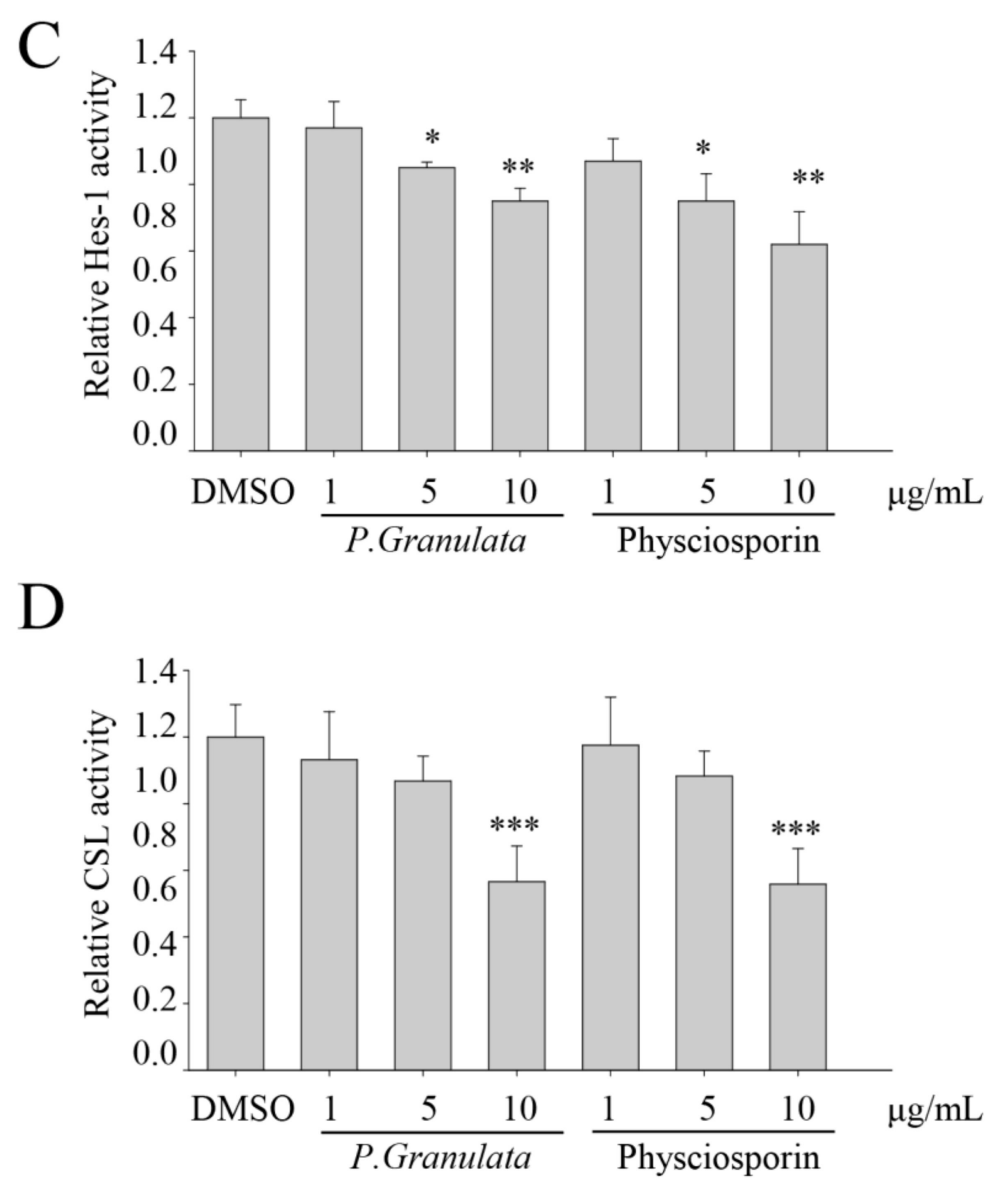
Effects of crude acetone extracts of *Pseudocyphellaria granulata* and physciosporin on transcription factor activity related to cancer stemness. (**A**–**D**) Relative activities of promoters related to Wnt/β-catenin, Sonic hedgehog, and Notch signaling pathways. HEK293T cells were co-transfected with pRL-TK (*Renilla*), pTOPFLASH (**A**), pGli-luc (**B**), pHES-luc (**C**), or pCSL-luc (**D**) reporter plasmids (firefly). After 12 h, transfected cells were treated with crude extracts of *P. granulata* or physciosporin and incubated for an additional 48 h. Relative firefly luciferase activity was compared between extract-, drug-, and DMSO-treated samples. Quantitative data were obtained from at least two independent experiments. Data are means ± standard error of the mean (SEM), and statistical analysis was performed by one-way ANOVA. * *p* < 0.05; ** *p* < 0.01; *** *p* < 0.001 vs. DMSO-treated HEK293T cells transfected with the target reporter plasmid.

To further investigate the target signaling pathways involved in the inhibition of cancer stemness by physciosporin, we performed reporter assays in HEK293T cells transfected with plasmids encoding gene-conjugated firefly luciferase: TOPFLASH-luc, Gli-luc, Hes1-luc, and CSL-luc. Hes1 targets Notch ligands, such as Dll1, Jagged1 (Jag1), and Neurogenin-2 [34,35]; Hes1-luc activity was significantly downregulated by crude extract of *P. granulata* or physciosporin at concentrations of both 5 and 10 µg/mL (Figure 4C). Gli and CSL are the main transcription factors of the Sonic hedgehog (SHH) and Notch signaling pathways, respectively; Gli-luc and CSL-luc activities were downregulated by *P. granulata* extract or physciosporin at 10 µg/mL (Figure 4B,D). TOPFLASH-luc examined β-catenin-mediated transcriptional activation [36,37]; however, TOPFLASH-luc activity did not exhibit any significant change after either treatment (Figure 4A). These data suggest that the SHH and Notch signaling pathways may be involved in the reduction of CRC stemness by physciosporin.

### 3.5. Inhibition of Spheroid Formation by Physciosporin was Abolished in CSC221 Cells Co-Overexpressing Gli1/2 and ΔEN1

**Figure 5:**
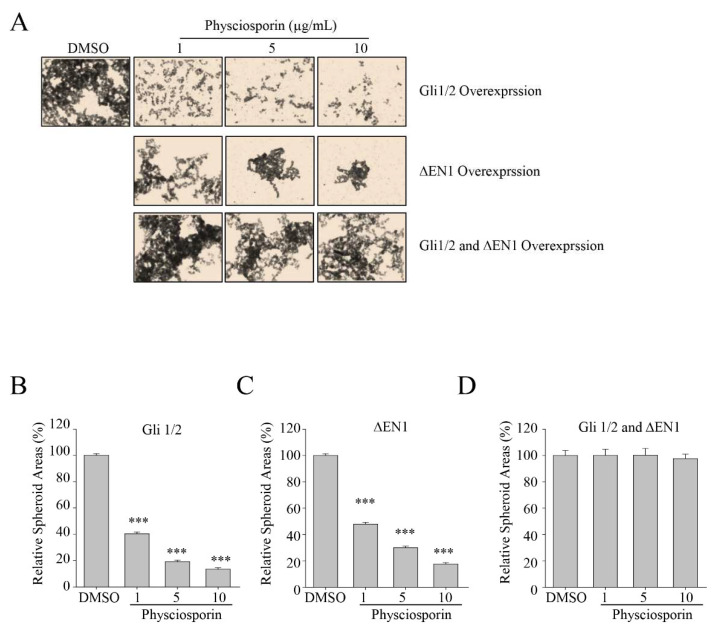
Effects of self-renewal potential of physciosporin on CSC221 cells overexpressing Gli1/2 and/or ΔEN1. (**A**) Representative images of spheroid formation of CSC221 cells overexpressing Gli1/2, ΔEN1, or both Gli1/2 and ΔEN1, treated with DMSO or various concentrations of physciosporin for 14 days. (**B**–**D**) Quantitative analysis of spheroid areas formed by CSC221 cells overexpressing Gli1/2 (**B**), ΔEN1 (**C**), or both Gli1/2 and ΔEN1 (**D**), treated with various concentrations of physciosporin. Quantitative data were obtained from three independent experiments, *n* = 3. Data are means ± standard error of the mean (SEM), and statistical analysis was performed by one-way ANOVA. *** *p* < 0.001 vs. DMSO-treated CSC221 cells overexpressing the same protein(s).

To confirm the involvement of the SHH and Notch signaling pathways in physciosporin-induced reduction of colon cancer cells, we subjected CSC221 cells overexpressing Gli1/2 and/or ΔEN1 to spheroid assays with exposure to physciosporin for 14 days (Figure 5). Overexpression of Gli1/2 and ΔEN1 activates SHH and Notch signaling, respectively. Physciosporin significantly suppressed spheroid formation in a dose-dependent manner in cells overexpressing either protein individually (Figure 5A–C) but exerted no inhibitory activity on cells co-overexpressing both Gli1/2 and ΔEN1 (Figure 5A,D), providing further confirmation that physciosporin suppresses colon cancer cell stemness through the SHH and Notch signaling pathways.

## 4. Discussion

Our previous findings showed that physciosporin can inhibit the cell motility of lung cancer cells and suppress the proliferation, motility, and tumorigenesis of colorectal cancer cells [26,27]. In this study, we expanded these findings as follows: (1) Physciosporin isolated from *P. granulata* exerted cytotoxic effects on CSC221 cells at 50 µg/mL. Crude extract of *P. granulata* exerted a much more potent cytotoxicity than pure physciosporin at 25 and 50 µg/mL. (2) At non-toxic concentrations, crude extract of *P. granulata* or physciosporin significantly decreased colony formation in soft agar and decreased spheroid formation by CSC221 cells. (3) Physciosporin also decreased spheroid formation by DLD1, Caco2, and HT29 cell lines. (4) Both protein and mRNA levels of *ALDH1* were sharply downregulated by treatment of crude extract and physciosporin. (5) Physciosporin decreased the transcriptional activity of the Gli, Hes1, and CSL promoter in reporter assays. (6) Physciosporin suppressed colon cancer cell stemness through the Sonic hedgehog and Notch signaling pathway.

The Notch signaling pathway plays a critical role in self-renewal and cell-fate decisions in undifferentiated pluripotent cells. Notch receptors are single-pass trans-membrane proteins with functional extracellular domains [17,22,38,39]. Interaction between Notch and its ligand initiates a signaling cascade that regulates differentiation, proliferation, and apoptosis. In our study, physciosporin in *P. granulata* extract blocked expression of Hes-1. Through this, acetone extract of *P. granulata* and physciosporin could block upstream signaling of the Notch pathway, causing the activity of the downstream factor Hes-1 to decrease.

We also observed that downstream targets of the Wnt/β-catenin pathway, such as CD44 and Lgr-5, were downregulated by treatment with acetone extract or physciosporin (Figure 3D,E); however, TOPFLASH-luc activity did not change significantly (Figure 4A). These results suggest that Notch might act downstream of Wnt/β-catenin in intestinal self-renewal, as well as in the promotion of proliferation in adenomas and adenocarcinomas of the intestine [17,20,22,38,39]. Recently, we reported that physciosporin suppresses the growth and motility of CRC cells cultured on 2D [27]. We showed that physciosporin downregulated β-catenin and its downstream target genes cyclin D1 and c-myc at 1.6 μg/mL. However, it was found that the expression of β-catenin, cyclin D1, and c-myc were not decreased by 1-μg/mL physciosporin treatment under spheroid formation condition (data not shown).

Hedgehog signaling is an important regulator of stem cell activity, stimulating self-renewal and proliferation of stem cells in various tissues [18,40,41]. Our previous study demonstrated that tumidulin isolated from *Niebla* sp. reduces spheroid formation in CSC221, DLD1, and HT29 cells at 5 μg/mL through the decrease of the transcriptional activity of Gli promoter via downregulation of Gli1, Gli2, and Smoothened (SMO) protein levels, which suggested that the SHH signaling pathway is one of target pathways involved in the inhibitory activity of tumidulin [28]. In this study, we found that acetone extract of *P. granulata* and physciosporin blocked the proliferation and self-renewal potential of CSC221 human colon cancer stem cells by suppressing Hedgehog signaling. qRT-PCR and promoter activity assays revealed that the upstream (Gli-1, Gli-2, and SMO) and downstream (Bmi-1) signals of the Sonic hedgehog pathway (Figure 3D,E) were significantly decreased by treatment with acetone extract and physciosporin. However, the upstream signals were also affected by these treatments, as demonstrated in cells overexpressing an upstream signaling factor of the Sonic hedgehog pathway (Gli1/2 overexpressed): In these cells, mRNAs encoding stemness factors were increased by over 30% relative to normal cells. Furthermore, the suppression of stemness factors by acetone extract and physciosporin was not completely compensated by overexpression of Gli1/2 because Notch signals were also significantly decreased by acetone extract and physciosporin.

The results of this study suggest that physciosporin plays a major role in the regulation of self-renewal and proliferation of CSCs. In this regard, our findings provide novel insight into the anti-cancer activity of lichen species.

## 5. Conclusions

In summary, physciosporin inhibited the spheroid formation in CRC cells and downregulated cancer stemness markers in in both protein and mRNA levels. Physciosporin suppresses the colon cancer cell stemness through the Sonic hedgehog and Notch signaling pathway.
