# Correction: Yang et al. Lichen Secondary Metabolite Physciosporin Decreases the Stemness Potential of Colorectal Cancer Cells. *Biomolecules* 2019, *9*, 797

**DOI:** 10.3390/biom14091085

**Published:** 2024-08-29

**Authors:** Yi Yang, Thanh Thi Nguyen, Iris Pereira, Jae-Seoun Hur, Hangun Kim

**Affiliations:** 1 College of Pharmacy, Sunchon National University, 255 Jungang-ro, Sunchon, Jeonnam 57922, Republic of Korea; yangyi_520@hotmail.com (Y.Y.); thanhbluesky21@gmail.com (T.T.N.); 2 Korean Lichen Research Institute, Sunchon National University, 255 Jungang-ro, Sunchon, Jeonnam 57922, Republic of Korea; jshur1@sunchon.ac.kr; 3 Department of Pharmacology, Chonnam National University Medical School, 160 Baekseo-ro, Dong-gu, Gwangju 61469, Republic of Korea; 4 Faculty of Natural Science and Technology, Tay Nguyen University, Buon Ma Thout 630000, Vietnam; 5 Institute of Biological Sciences, Universidad de Talca, Talca 747-721, Chile; ipereira@utalca.cl

In the original publication [1], there were overlaps in Figures 1D and 5A as published. In the first row of Figure 1D, images 3 and 4 were not correct; therefore, they have been replaced. In the first row of Figure 5A, image 3 as well as the third row in image 2 are not correct; therefore, they have also been replaced.

In contrast to migration, invasion, and colony formation assays, spheroids in the spheroid formation assay tend to form with aggregation in center area of the low-attachment 24-well plate. In some cases, the number of spheroids is limited or too large. Therefore, in order to quantify this, image acquisition with some overlapping areas is inevitable. And this is the reason for the overlapping images.

The corrected Figure 1D and Figure 5A appear below.

The authors state that the scientific conclusions are unaffected. This correction was approved by the Academic Editor. The original publication has also been updated.

The authors apologize for any inconveniences caused and state that the scientific conclusions are unaffected.

## Figures and Tables

**Figure 1:**
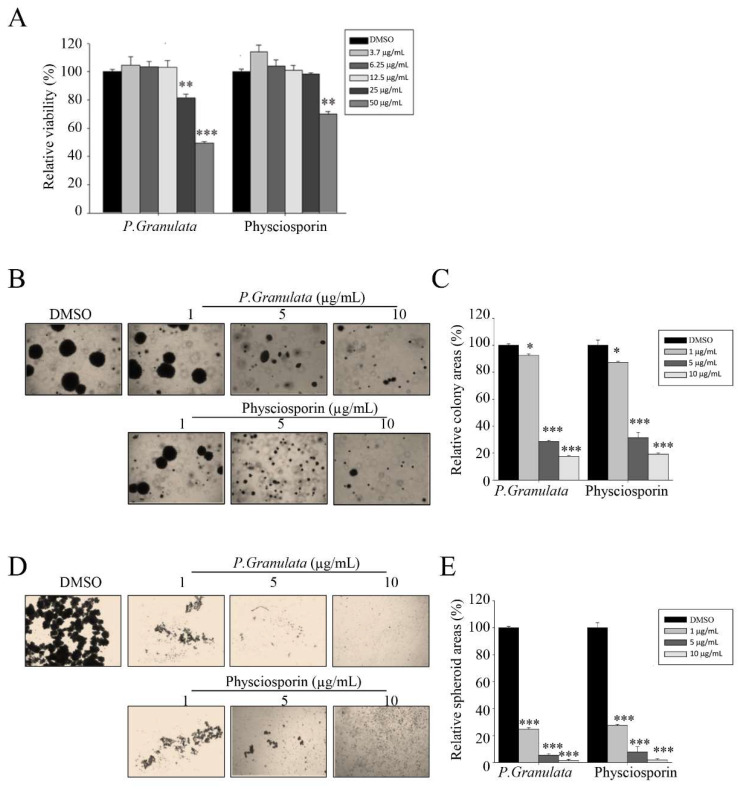
Acetone extracts of *Pseudocyphellaria granulata* and physciosporin inhibit CRC221 cell stemness. (**A**) Relative viability of cells treated with acetone extract of *P. granulata* or pure physciosporin. CSC221 cells were treated with crude extract or physciosporin at concentrations ranging from 3.7 to 50 µg/mL for 48 h, and cell viability was measured by MTT assay. (**B**,**C**) Soft agar colony-formation assay of CSC221 cells treated with *P. granulata* crude extract or physciosporin (**B**), and quantification of the percent colony area in each group (**C**). (**D**,**E**) Representative images of spheroid formation of CSC221 cells treated with *P. granulata* crude extract and single-compound physciosporin for 14 days (**D**), and quantitative analysis of the number of spheroids following each treatment (**E**). Quantitative data were obtained from three independent experiments (*n* = 3). Data are means ± standard error of the mean (SEM), and statistical analysis was performed by one-way ANOVA. * *p* < 0.05; ** *p* < 0.01; *** *p* < 0.001 vs. CSC221 cells treated with DMSO.

**Figure 5:**
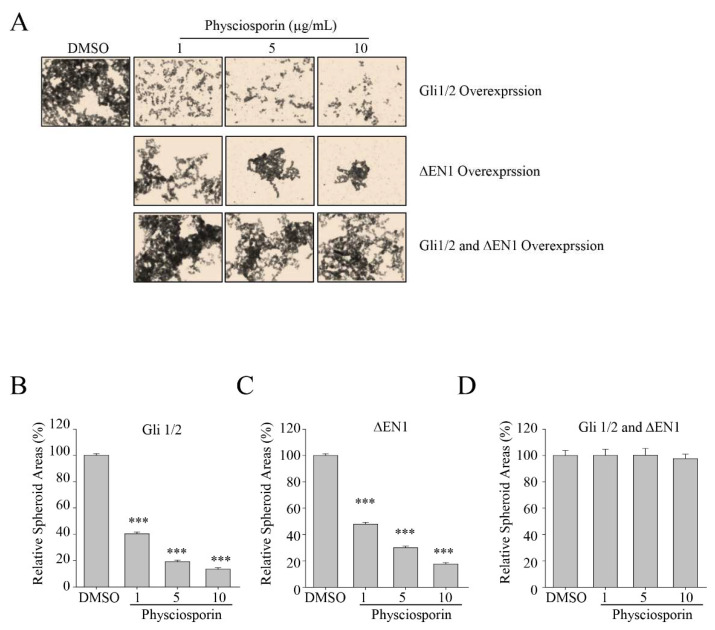
Effects of self-renewal potential of physciosporin on CSC221 cells overexpressing Gli1/2 and/or ΔEN1. (**A**) Representative images of spheroid formation of CSC221 cells overexpressing Gli1/2, ΔEN1, or both Gli1/2 and ΔEN1, treated with DMSO or various concentrations of physciosporin for 14 days. (**B**–**D**) Quantitative analysis of spheroid areas formed by CSC221 cells overexpressing Gli1/2 (**B**), ΔEN1 (**C**), or both Gli1/2 and ΔEN1 (**D**), treated with various concentrations of physciosporin. Quantitative data were obtained from three independent experiments, *n* = 3. Data are means ± standard error of the mean (SEM), and statistical analysis was performed by one-way ANOVA. *** *p* < 0.001 vs. DMSO-treated CSC221 cells overexpressing the same protein(s).
